# Diseases of the Heart and Chest

**Published:** 1835-07-01

**Authors:** 


1. The Pathology and Diagnosis of Diseases of the Chest; illustrated especially by a Rational Exposition of their Physical Signs. With New Researches on the Sounds of the Heart. By *Charles J. B. Williams*, M.D. &c. Third Edition, much enlarged. Octavo, pp. 209, with two Plates, and Tables.

2. Appendix to the Second Edition of a Treatise on the Diseases of the Heart and Great Vessels, &c. By *J. Hope*, M.D. F.R.S. Assistant Physician to St. George's Hospital, &c. 1835.*

It is gratifying to observe the steady progress in public estimation made by the study of the physical signs of diseases of the chest. It is but a few, a very few years, since the advocates of auscultation were ridiculed in private and in public—in societies and journals. Those who laughed at the inutile lignum, were so blind as not to perceive their own absurd position, absurd, because they voluntarily closed the ears which Nature gave, against the sounds she uttered. They employed the nose, the eyes, perhaps the tongue,† because their forefathers had set them the example. But the same reason induced them to reject the sense of hearing—a happy thought, exactly similar to that of the celebrated sheep of Panurgus, who jumped into the sea because their leader fell in.

Such opposition, based on prejudice, and indefensible by reason and by common sense, could not possibly withstand the progress of knowledge. Such a mop could not dry up such an Atlantic. All public opposition, in fact, has ceased, and become as completely a matter of history as the equally wise, but more malignant attacks on Galileo, or the loud and general clamour against Harvey.

There can be no question that the first pretensions of the auscultators, or of many of them, were extravagant. They affected a precision which is perfectly unattainable, and depended on auscultation for more than it can offer. Such enthusiasm was natural, and may be excused; but we fear that, even now, there are some who make the stethoscope an instrument of delusion, possibly to themselves, but certainly to their patients. Happily, such conscious or unconscious quackery is limited to a few, and those the least respectable members of the profession, and the better informed and better principled are earnest in exposing the indecent fraud.

Dr. Williams adopts that sober view of the value of the physical signs of disease of the thoracic organs, which all men of experience and honesty must sanction. We cordially agree with him in the scope and tenor of the following remarks, contained in the preface to the edition now before us.

---

* This Appendix arrived as we were completing the present article. The second edition of the work itself has not yet reached us.—Eds.

† It is not very long since the stools were tasted. The urine may occasionally be so still.

"Within the last few years (he says), auscultation has been rapidly advanced in the estimation of the profession; and I think this is much to be ascribed to the more rational and moderate manner in which its pretensions have been urged. Instead of being blindly put forth as infallibly indicating such and such diseases, its signs are now appreciated only as they inform us of certain physical changes in the organs within the chest; whilst the aid of our other senses, a due attention to the general symptoms, and a careful reflection on all these evidences, are also required, to enlighten us in our study of disease, and to suggest remedies for its removal. It is with a view to prevent the student from relying too exclusively on the physical signs, that I have in the present edition added a short summary of the general symptoms of each disease, which in very many instances are more important in guiding our practice, than the local signs."

We were the first to impress on the profession of Great Britain the imperative necessity of cultivating an acquaintance with auscultation and percussion—we have since laboured sedulously, and probably not altogether unsuccessfully, in the field—yet we do not hesitate to admit that the estimate of our author is a just one, and to add our belief, that the mere auscultator is as dangerous a personage as could be placed in a sick room. We seldom pass a week without observing some pregnant illustration of the blunders of such gentlemen.

We are induced to notice this, a third edition, because it really contains new matter. That new matter is judiciously and specifically stated by the author, a piece of information equally valuable to the reader and the critic. The additions are actually as follow:—The sections *On the Ocular and Manual Examination of the Chest; On Expectoration; On Encephaloid Disease, Melanosis, &c.; On Diseases of the Bronchial Glands;* and the whole of Part III., *On the Auscultation of the Heart*—are new; and large additions have been made to the sections on *Bronchitis, Peripneumony, Pleurisy, Pneumothorax,* and *Phthisis.* This new matter will alone receive our notice, and to this we therefore proceed.

### I. OCULAR AND MANUAL EXAMINATION OF THE CHEST.

To the general principles on which percussion and auscultation are founded, it would be almost impertinent to refer. Such principles, the A B C of science, we must now suppose familiar to our reader. Yet, if any be still unacquainted with them, we refer them to the various elementary works upon the subject, some of which all members of the profession should possess, and not only possess but diligently study. Yet the ocular and manual examination of the chest may be considered as a step preliminary to that of percussion or of auscultation. It is less exact than they are, but it offers data frequently important, and always useful.

In a well-formed and a healthy chest, both sides should be equally raised and depressed in the act of respiration. That of inspiration is of a compound character—partly affected by the elevation of the ribs, partly by the contraction and descent of the diaphragm. If, then, the chest be unequally expanded—if one side rise more or less than the other—or if respiration be carried on exclusively by the ribs or diaphragm, it is obvious that there is a deviation from the healthy condition of the thoracic apparatus, and the cause of that deviation must be sought. It may be such as to be obvious at once to the eye, or it may require the discriminating assistance of the touch. Let us then understand that the action of the chest presents two important physical deviations from the natural standard;—in the first, the respiration is too exclusively performed by the ribs, or too exclusively carried on by the diaphragm; in the second, there is inequality in the motion of the respective sides of the thorax. The indications derived from the former are the least valuable, and may be first disposed of.

"If any disorder," says Dr. Williams, "impede the free descent of the diaphragm, whether it be pain of this muscle or its coverings, or of some of the viscera below, or whether it be pressure on its under surface, as from abdominal dropsy, tumours, or pregnancy, an increased task will then devolve on the ribs and their muscles, and the respiration being performed principally by the heaving of the chest, is called *thoracic*. This character of respiration is obvious to the eye: and one hand applied lightly, but in close contact, on the chest, and the other on the abdomen, equally perceive it: it becomes then the next matter of inquiry, which of the above mentioned causes is present. Again, in the converse case, which belongs more closely to our subject, pain or increased sensibility of the parietes of the chest, or of its more superficial contents, ossification of the cartilages of the ribs, and occasionally certain changes in the lungs themselves, which will be afterwards considered, make the respiration *diaphragmatic*, or *abdominal*, the ribs remaining comparatively immobile. The diseases of the chest which may render the respiration chiefly abdominal, are pleurodyne, inflammation of the costal and upper pulmonary pleura, and occasionally induration of the lung by hepatisation or tubercles. Of those which render the respiration chiefly thoracic, besides diaphragmatic pleurisy, spasmodic asthma may be reckoned, in which the diaphragm, overcome by the superior force of the bronchial muscles spasmodically contracted, is permanently drawn into the chest, causing a remarkable hollow at the scrobiculus cordis, whilst the respiration is carried on by the intercostal muscles, and others which assist them in supplemental respiratory efforts." 13.

It is obvious that several and opposite causes conspire to render the respiration thoracic or abdominal. In the mental process of investigation, the physician, informed by the physical signs which of the two unnatural conditions exists, passes rapidly in review the causes of both, and proceeds to determine by the analytical examination of other symptoms, which of those causes is the true one. And this it is which constitutes the value of any general symbol of disease. It circumscribes and directs inquiry.

By the way, the rigid critic might reasonably take exception against the bold assumption of our author, in reference to the pathology of spasmodic asthma. We are not sure, even though Dr. Williams be so, that in that complaint the "bronchial muscles" exert a force superior to the diaphragm, and forcibly draw it into the thorax. This is, at the best, a pretty piece of theory, and in a work devoted to the most precise and demonstrable division of symptomatology, the less of theory, perhaps, the better.

A difference of action in the two sides of the chest furnishes very important indications. The want of correspondence may be in form or motion, or indeed in both. As both lungs are seldom affected at the same time, except in phthisis, a sign which at once and obviously determines something unnatural in one, must evidently be valuable to the surgeon or physician.

Any disease, as our author observes, which interferes with the respiratory act, and affecting chiefly one side, will produce an inequality obvious to sight and feeling. For this mode of examination, the patient should be placed with his chest exposed in a good light opposite to the observer, who attentively surveys the chest, and further corrects his estimate of its form and motions, by feeling with his two hands the simultaneous motions of corresponding parts on the two sides, during increased as well as ordinary acts of respiration. To determine the comparative size of the two sides of the chest, the more accurate method of measurement from the spinous process of a vertebra to the sternum may be sometimes employed. Care must be taken that the string or tape be passed over corresponding parts; and it must be held in recollection, that in healthy persons the right side is almost always slightly larger than the left.

"Now," continues Dr. Williams, "it is obvious, that any inequality or irregularity in the form or movement of the chest will imply some disease; and, with a little further attention, the same method of examination will give some general knowledge of its seat and character. Thus, if one side appears to be immobile, with a sharp pain or stitch, but without alteration of size, it may be suspected that a pleurodyne or a recent pleurisy is the disease, and prevents the respiratory movements by the pain which it would cause in the affected part. If to the immobility of the side is joined an unnatural fulness, perceptible to the eye and to mensuration, there is perhaps effusion into the pleura, as in advanced states of pleurisy, empyema, hydrothorax, and pneumothorax. If a contraction of the side is joined to the defect of movement, adhesions or the reabsorption of a pleuritic effusion of some standing, is the probable cause. If one side does not partake in the respiratory act, and yet there is neither pain nor alteration of size, it is likely that the corresponding lungs may be hepatised, or otherwise obstructed.

The preceding signs are connected principally with the middle and lower parts of the chest. Irregularities in the movements and shape of the upper regions are perhaps more characteristic. Thus tubercular disease rarely exists to a considerable extent without diminishing the motion of the upper ribs of the affected side; and adhering, as the diseased lung often is, to the walls of the chest, it not unfrequently causes an angularity and want of symmetry, which are very characteristic. Opposed to this is the effect of pulmonary emphysema, which gives a full and unnaturally rounded appearance to the upper parts of the chest, whilst, both by sight and tact, it can be perceived that the chest does not rise and fall to its natural capacity." 15.

Dr. Williams remarks, and properly remarks, that the preceding signs are too vague and indecisive to do more than lead the way to accurate discrimination. Yet they serve as sign-posts, and though they may not take us to our journey's end, they tell us the shortest and the safest road to it. There is another advantage in attending to signs of this description. They induce a habit of minute and accurate investigation, a habit which is often productive of the most important consequences. The surgeon who is conversant with percussion and with auscultation, will frequently detect a tumor or an aneurism, which others have omitted to observe, because they have omitted to examine.

The exploration of the condition of the intercostal spaces by the fingers is occasionally productive of advantage. The pulsations of the heart and of aneurismal tumors may be thus distinguished. We have found this kind of examination of service in cases of acute pleural inflammation. When this exists, the tenderness on pressure of the intercostal spaces is occasionally great, and this is at times a valuable indication.

Of course, in practising ocular and manual examination of the chest, the physician must not commit the blunder of mistaking for disease actually in progress, those deformities produced by curvature of the spine, or by improper nursing. The effects of tight lacing in females become also obvious to the eye, in the permanently contracted and less mobile state of the lower parts of the chest, and in the increased heaving of the upper ribs.

Such is the section on that preliminary examination of the chest which should always, if possible, precede any more minute investigations.

### On Expectoration.

There are two ways of studying disease. The first and best is the analytical—that by which we unravel symptoms, and trace them back to the physical lesions by which they are occasioned. This is the philosophical method, and this is the very basis of all sound and satisfactory medical science. But it is frequently advantageous to neglect this exact and laborious train of reasoning, and to erect for the occasion particular symptoms into substantive diseases. The patient, for instance, has pain in the back. The philosophic surgeon investigates the nature of the accompanying phenomena, and endeavours to determine on what that pain may really depend. Effecting this, he directs his attention and his remedies to the cause and not to the mere consequence, and this is the aim and the triumph of science. But the proceeding may be usefully reversed, usefully by him who is conversant with the better and the nobler mode of study. He may look on pain of the back, per se, as a morbid state, generalize the circumstances under whioh it occurs, arrange the various conditions that produce it, and determine the remedies most calculated to relieve it. It is the combination of the knowledge derived from both these sources, that constitutes the accomplished practitioner.

These remarks were occasioned and may perhaps appear borne out by the short section on a symptom of disease of the thoracic organs—expectoration. That this must be a symptom of many and of very various conditions we need hardly say. Yet even as a symptom it assumes importance, from the states it represents, and the treatment it requires. Let us hear what Dr. Williams has to say upon the subject. He first considers physiologically the act, and then pathologically the matter of expectoration.

"The act," says he, "of expectoration is one of the instances of combined movement in the respiratory machine, which by an admirable and harmonious consent between its numerous muscles, unerringly produces such a variety of actions. The function of respiration is of such vital importance, that accumulations or effusions that obstruct it, endanger life itself. The structure of the bronchial tree contributes greatly to the easy removal of any superfluous matter in it that might cause such obstruction, for the sum of the area of its branches being considerably greater than that of its trunk, the trachea, the air commonly finds easy entrance into the air cells, and on its more rapid return in expiration, carries with it the superfluous matter. Thus ordinary respiration tends to prevent, in spite of gravitation, any accumulation in the air tubes; but the excretion is more completely effected by coughing, and special efforts of expectoration. These consist of a quick and forcible expiration, preceded by a deep inspiration, and accompanied with a constriction of the larynx and trachea, the effect of which is to bring any superfluous matter into positions, from which the air, forcibly expired, drives it through the glottis. It is worthy of remark that expectoration cannot effectually take place without a previous full inspiration by which air is carried beyond the accumulating matter; hence, when this is prevented, either by weakness of the respiratory powers, or by the impermeability of the bronchial tubes, the excretion is suppressed. The first of these causes of obstructed respiration is exemplified in adynamic fevers, which may thus prove fatal; the second occurs in pneumonia, in the stage of hepatisation, and if extensive, must lead to a fatal obstruction of the respiratory function. They probably occur together towards the fatal termination of bronchitis, phthisis, and other severe diseases of the lungs." 45.

The matter expectorated is surely deserving of serious attention, exhibiting as a secretion the state of the secerning parts. Dr. Williams laments, and not without some justice, the imperfect information too often evinced by practitioners on this point. We have often seen the genuine tuberculous expectoration considered as of little moment—and, commonly, the presence of pus in the expectoration is deemed a sure proof of the lungs being "diseased." Dr. W. too remarks, that the pathognomonic sputa of peripneumony, and the well-marked secretion of acute bronchitis are hardly recognized, indeed commonly mistaken. All Dr. Williams has to say is soon said.

"The natural secretion of the bronchial mucous membrane is a colourless liquid of somewhat glutinous quality, like a thin solution of gum arabic. It does not much differ in composition from the serum of the blood, and owes its viscidity to an animal substance, which Dr. Pearson, Dr. Bostock, and Berzelius, concur in considering an imperfectly coagulated albumen. This secretion is the basis of most of the varieties of expectorated matter; but unhappily our present knowledge of animal chemistry does not enable us to discover the precise nature of the changes in composition which produce these varieties. All that we learn is, that albumen in different forms and proportions is present; for whether the expectoration be mucus, serum, pus, tuberculous matter, or coagulable lymph, the chemist can discover in these but scarcely discernible varieties of the same principle. There is a considerable variation in the proportion of saline matter in different kinds of expectoration; and on this depends a distinction, formerly much insisted on, by means of the salt or sweet taste. This criterion certainly fails to distinguish pus from mucus; but I think that an excess of saline matter may be taken as a sign of inflammatory action in the mucous membrane.

It is by its mechanical and visible conditions, however, that expectorated matter is most distinctly characterized; and to examine these fairly, the entire sputa should be collected in one or more convenient vessels of white ware or glass, in which their quantity, colour, and consistence, can be minutely scrutinized." 46.

We think it would have been well if our author had dwelt a little more at large on the varieties of the expectoration. He has enumerated wants but not supplied them—a tantalizing process.

### Encephaloid Disease—Melanosis, &c. of the Lungs.

Dr. Williams offers little on these diseases to detain us. He states that melanosis, encephaloid, or medullary sarcoma, fungus hæmatodes, and scirrhus, are occasionally developed in the pulmonary tissue; and they may occur in a circumscribed form, or diffused through a considerable extent of texture. They would then produce the same physical signs as tuberculous disease of analogous extent, and could be distinguished from this only by the general symptoms, and the absence of the constitutional indications of tubercle.

"These diseases," he proceeds, "usually prove fatal, either by encroachment on the function of the lungs, or from being simultaneously developed in other organs (especially in the liver) before the process of softening and ulceration can be effected. To this, nevertheless, they certainly tend; for I have seen instances, and others are on record, of ulcerous cavities formed in melanose and encephaloid solidifications of the lung; and the expectoration, in one case of a black and red, and in the other of a streaky whitish and sanguinolent purilaginous matter, led to a suspicion of the nature of the diseases, before death. In such cases the stethoscope would indicate the gurgling and other signs of a cavern in the lung. The general signs of these affections are more commonly those of obstructed circulation, dropsy, &c., with oppressed breathing, than those of emaciation, common in tuberculous disease. The difference in the duration and progress of these several morbid productions sufficiently explain the variety in the symptoms; and I am disposed to think that these differences proceed in great measure from the mechanical condition and degree of vitality in which these matters are deposited from the blood, as I have endeavoured to point out analogous circumstances in the nature and changes of tubercle." 154.

We do not see any thing in Dr. Williams's brief observations on the structure of encephaloid disease, or of melanosis, that need occupy our attention. We may mention, par parenthèse, an instance of complete conversion of one lung into encephaloid disease which we witnessed some years ago at St. George's Hospital. There was measurable contraction of one side of the chest, and this was thought to be the consequence of former pleurisy. But the patient became hectic, had symptoms which resembled phthisis, and died. On dissection the disease was such as we have mentioned.

With our author's observations on the black matter found in the lungs of the older inhabitants of large towns, we leave the present section.

"The black matter which is found in the lungs of the older inhabitants of large towns, is a carbonaceous matter, and beyond doubt is derived from the soot inhaled in the air, as first supposed by Dr. Pearson. Its deposition and permanency in the tissue of the lungs, is a proof, not (as Majendie maintained in the analogous case of the carbonaceous matter of tattoed skins, and of the insoluble oxide in persons coloured by the internal use of nitrate of silver) that there are no textural reparation and absorption, but that this absorption cannot act on insoluble solid matter. I believe that this black matter finds access to the pulmonary texture principally through abrasions, softenings, or other lesions of the bronchial mucous membrane, slight injuries of this kind being common accompaniments of an ordinary cold or cough. This coaly dust does not appear generally to produce any injury to the function of the lung, but getting into any corners out of the immediate sweep of the circulation, such as in the angles of the lobules, or the sides of large vessels, in cicatrices of old lesions, and in the bronchial glands, it remains slowly accumulating until death, or until it is carried off, in expectoration, by some pulmonary disease. But there are some curious instances on record, in which this accumulation has taken place so rapidly and extensively as to infringe on the function of the lung, producing œdema and a black consolidation of the tissue, which tends to ulceration and the formation of cavities. A case has been described by my lamented friend, Dr. J. E. Gregory,* in which the habitual inhalation of the dust of a coal mine had produced a disease of this kind, accompanied with the cachectic anasarcous state consequent on the obstruction to the pulmonary function. Other cases are related by Dr. W. Thomson,† in which a similar affection could be traced to continued employment by the light of lamps which give out much sooty smoke. In these instances an additional cause of pulmonary disease seems to be required to produce the black infiltration in the manner which I have mentioned; and this cause was presented either in prior bronchial complaints, or in the occupation of the individuals as stonemasons, which of itself frequently produces severe lesions of the bronchial membrane, and even of the pulmonary tissue. In Dr. Gregory's case the coal dust was the mechanical irritant as well as the colouring matter." 156.

### Diseases of the Bronchial Glands.

Dr. Williams has not much to offer upon this head. The bronchial glands are occasionally enlarged from inflammatory action, and Laennec in a few cases, found them the seat of abscess. They are frequently the seat of tuberculous deposit, which Dr. Williams has seen exclusively affect them. When thus tuberculous, they sometimes attain the size of a pullet's egg, and by pressure on the trachea or bronchi, or on the blood-vessels, induce dyspnœa and obstructed circulation. Dr. Carswell considers this to be not uncommon in children; and he would ascribe to it the dyspnœa occurring in them, without signs of other disease, when the chest continues to be sonorous on percussion, and the respiration every where audible though weak. Tuberculous matter in the bronchial glands is liable to the softening, and to the drying and cretaceous conversion which affect this deposite in the lungs. The softened matter may be evacuated through the bronchi, leaving a fistulous cavity; and Guersent describes this as not an uncommon case in children. Dr. Carswell notices an instance of a child, in which this softening and ulceration proved fatal, by opening a branch of the pulmonary artery. But sometimes calcareous matter in the bronchial glands would seem to be formed independently of any previous tuberculous alteration.

Encephaloid disease may originate in these glands, and extend from them to the lungs.

Of the physical signs of alterations in the bronchial glands we think we may safely be excused from speaking. They are too vague to require notice, or, in practice, to deserve attention.

---

* Edinb. Med. and Surg. Journal, No. 109.

† In a paper read to the Medical Section of the British Association, Edinburgh, 1834. See also a paper by Mr. Graham of Glasgow, on Charcoal in the Lungs.—*Edinb. Med. and Surg. Journal, No.* 121.

We may now proceed to the part of this edition which is most deserving of consideration. It is that which is devoted to the auscultation of the heart. Our readers must be aware that many experiments have been lately made in order to determine, if that be possible, the characters and causes of the sounds which accompany the action of the heart. The experimenters have been, perhaps, as numerous as the experiments, and the conclusions have been almost as various as the experimenters. Yet this is an inducement to the man of ingenuity and perseverance, to endeavour to disentangle the knot, which would seem to have baffled so many attempts at its untying.

### Auscultation of the Heart.

We are induced to devote some little space to this interesting subject, because the diseases of the heart, though frequent, are occasionally obscure, and not uncommonly mistaken: and because, though much is yet uncertain, something would seem to be determined. For, as Dr. Williams judiciously observes, it may be useful to separate and appreciate the points that really are certain and useful, from those that are doubtful or ill-defined, both with a view to make our present knowledge safely available to the student, and to guide future inquirers in further investigations. Dr. Williams pays a well-merited tribute to the labours of Dr. Hope, a gentleman whose industry and talents must eventually confer on him a high degree of professional eminence.

In order to enable the majority of our readers to comprehend the nature and the scope of the experiments to which we shall presently advert, and the conclusions which might seem to be fairly drawn from them, we must venture to state as succinctly as possible the sound produced by the heart in health, and heard on applying the ear to the chest. We think we cannot do this better than in the words of Dr. Williams. We shall therefore introduce them here.

"On applying the ear to the region of the heart in a healthy person, a sound is heard at each pulsation, followed by an interval of silence. This sound is double; consisting of a dull, slow sound, immediately followed by a short quick one. The second or short one accompanies the ventricular diastole. Laennec rates the relative duration of these, in each ordinary pulsation, to be as follows: the first sound, two-fourths; the second sound, one-fourth, or a little more: the interval of silence, one-fourth, or a little less. These sounds are naturally most distinct in the space between the cartilages of the fourth and seventh ribs of the left side, and on the lower part of the sternum; the former part corresponding with the left, and the latter with the right side of the heart. Beyond this space the sounds are rarely distinct, in persons of good proportion and middling stoutness. In thin subjects, they are heard over the whole front of the chest, and sometimes reach to the left dorsal, and rarely to the right dorsal regions. I have very commonly found them audible through the vertebræ between the scapulæ.

Simultaneously with the first, or systolic sound, an impulse or shock is communicated to the stethoscope. It is most perceptible at and between the cartilages of the fifth and sixth ribs, where it may be felt by the hand; but the stethoscope commonly renders it sensible in lean persons over the whole præcordia, and if the sternum is short, in the epigastrium also." 161.

Thus, then, there are two sounds heard, and one impulse felt. Of the two sounds, the first is duller and of longer duration—the second clearer and of shorter duration. The impulse is simultaneous with the first sound, and that and the impulse are synchronous with the arterial pulse. These are facts which scarcely admit of dispute or doubt, though both, indeed, have been raised on them, demonstrative as they appear. But nothing can exceed the confusion that prevails with respect to the explanation of them. The second of the chapters in the present work, devoted to the auscultation of the heart, contains an inquiry into the nature of the motions and sounds of that organ, and displays, in a striking manner, the variety of hypotheses advanced, refuted, and re-advanced upon the subject. To this inquiry we shall devote some space; for our information is acquiring a degree of distinctness and precision, and it may be useful to point out in the pages of this Journal what is true, what is false, and what is still uncertain.

In order to render this article an useful one, it will be necessary to enter at some length into particulars—to review what has been done—to shew what still remains for us to do—to exhibit the various opinions that have been advanced—to display the fallacy of those which are fallacious—and to endeavour, as far as a periodical writer can, to prevent future experimenters and inquirers from going unnecessarily over the same ground, re-discovering the same truths, or re-inventing the same errors. We are necessarily compelled to indulge in extract rather than analysis on this occasion; for Dr. Williams has already composed an abbreviated summary of former facts, and the details of experiments and their results are scarcely susceptible of advantageous condensation.

First, then, we will enumerate, in Dr. Williams' words, the hypotheses that have distracted the stethoscopic world—and add the objections, reasonable or conclusive, that may be severally urged against them. The views (and, if the list is not absolutely perfect, it is sufficiently complete) are, seriatim, as follows:—

"1. M. Laennec. *a.* 1st sound, impulse, and pulse, caused by the ventricular systole. *b.* 2nd sound by the systole of the auricles.—*Remarks.* *a.* Generally admitted, and proved by various facts and experiments. *b.* Disproved by the fact noticed by Harvey and Haller, and confirmed by modern experiments, that the auricular contraction immediately precedes that of the ventricles: also by this fact, that both sounds sometimes continue after the auricles have ceased to contract.*

2. Mr. Turner.† 2nd sound produced by the falling back of the heart on the pericardium after the systole of the ventricles.—*Remark.* Disproved by the fact, that the sound continues when the heart pulsates out of the pericardium.

3. Dr. Corrigan.‡ *a.* Impulse and 1st sound caused by the rush of blood into the ventricles during the auricular systole. *b.* 2nd sound by the ventricular systole, which he considers to be instantaneous.—*Remarks.* *a.* Disproved by the clearly ascertained facts, that the 1st sound and impulse accompany the systole of the ventricles when the auricles have ceased to contract. *b.* Disproved clearly in large animals by the ventricular systole (which is not instantaneous),

* Dr. Hope's Experiments on Asses. See his Work, p. 36.
† Med.-Chir. Trans. Edin. vol. iii.
‡ Trans. of King's and Queen's Coll. of Phys. Ireland.

and the pulse of arteries near the heart, evidently preceding the 2nd sound;* and further disproved by several pathological phenomena.

4. Dr. David Williams.† 2nd sound caused by the flapping open of the auriculo-ventricular valves against the sides of the ventricles; these valves he supposes to be opened by the musculi papillares.—*Remark.* This is contrary to the received opinion of anatomists with respect to the functions of the auricular valves and musculi papillares, and there is no collateral argument to maintain so gratuitous an assumption.

5. M. Pigeaux.‡ *a.* 1st sound produced by the blood rushing into the ventricles at the moment of their diastole. *b.* 2nd sound by the collision of the blood against the walls of the aorta and pulmonary artery. *c.* The ventricles contract in a moment of silence before the 2nd sound. *d.* The intensity of the sounds proportioned to the force by which the blood is impelled.—*Remarks. a.* Opposed by the facts stated against 3 *a*; opposed also by many pathological facts, such as the occurrence of a murmur with the 1st sound, in case of diseased semilunar valves. *b.* Disproved by the fact, that the 2nd sound occurs distinctly *after* the pulse in the carotids, and therefore after that in the larger arteries. *c.* Opposed by the observation, that the 1st sound and ventricular systole occur together and correspond in duration. *d.* This is opposed by the morbid phenomena of dilatation of the ventricles, which always increases the first sound, and of hypertrophy, which diminishes both sounds.

6. M. Majendie.‖ 1st sound and 1st impulse produced by the ventricular systole impelling the apex with a shock against the walls of the chest between the fifth and sixth ribs; the 2nd sound and 2nd impulse by the blood, in refilling the ventricles at their diastole, forcing their parietes with a shock against the sternum.—*Remark.* Completely disproved by the fact, that both sounds continue when the heart does not touch the parietes of the chest.§

7. M. Rouanet.¶ *a.* 1st sound caused by the closing of the mitral and tricuspid valves against the auriculo-ventricular orifices during the ventricular systole. *b.* 2nd sound by the re-action of the blood in the arteries on the semilunar valves at the moment of the ventricular diastole.

8. Mr. H. Carlile.** *a.* 1st sound produced by the rush of blood into the arteries during the ventricular systole. *b.* 2nd sound by the re-action on the semilunar valves, as stated in *b* 7.

9. Dr. Hope. *a.* 1st sound and impulse, caused by the ventricular systole. *b.* 2nd sound and *back stroke,* or 2nd impulse, by the ventricular diastole. The natural as well as morbid sounds produced by the motions of the contained fluid." 165.

In order to proceed with any thing like clearness, we must now revert to

---

* Dr. Hope's Experiments, p. 31 of his work; and those of Mr. Carlile, Dublin Journal of Medical Science, vol. iv.

† Edinb. Med. and Surg. Journal, Oct. 1829.

‡ Arch. Génerales de Médecine, Juillet et Novembre, 1832.

‖ In Lectures delivered at the College of France, in 1834, reported in the Lancet, Feb. 1835.

§ See the Experiments of Dr. Hope (p. 30 *et seq.* of his work), of Bouillaud (Journ. Hebdom.), and my own, as described further on in the text. Many other facts might be stated as conclusive against this "last new view," but the above-named one seems to me quite sufficient.

¶ Journ. Hebdom. No. 97; also Mr. Bryan, Lancet, Sept. 1833, and M. Bouillaud, Journ. Hebdomad. 1834.

** Dublin Journal of Medical Science, vol. iv. The essay was likewise read at the Cambridge Meeting of the British Association.

the experiments performed by Dr. Hope, and detailed in his work on the Diseases of the Heart. We must content ourselves with stating the most important and most obvious deductions drawn from the experiments in question. For the details of the experiments themselves we must refer to the excellent work of Dr. Hope. It may be considered, then, as demonstrated:—

1st. That the auricles contract first, producing no sound.

2nd. That the auricular contraction is immediately followed by the ventricular systole, which is accompanied by the first or dull sound. This systole, by straightening the anterior convexity of the ventricles, brings their apex into forcible contact with the ribs, and thus is produced the impulse. This systole, by throwing an additional quantity of blood into the arteries, causes the arterial pulse, which in arteries near the heart is synchronous with the ventricular systole, but in those more distant succeeds it at an interval occupied by the transmission of the wave through the blood along the elastic tubes from the heart.

3rd. That the ventricular systole is immediately followed by the diastole, which is accompanied by the 2nd or short sound.

4th. That there is then an interval of rest, at the conclusion of which the auricles contract, and the series of motions is repeated as before.

These are facts. The concurrence of the first sound, impulse, and arterial pulse with the ventricular systole: and the absence of all appreciable sound on the contraction of the auricles are plain, obvious, undeniable facts, which all with eyes and ears may see and hear, in experiments on large animals. At many of those experiments we have been present, and for what has been stated we can personally vouch. The second sound has clearly nothing to do with the auricle, and it *appears* to co-exist with the ventricular diastole. Yet, so far as our personal satisfaction and belief go, the production of the second sound is involved in much more obscurity than that of the first.

A little consideration would shew even the most inconsiderate reader, that the *immediate* cause of the sounds of the heart continues unexplained. The first sound is manifestly connected with the systole of the ventricles, and depends upon it. But how is it produced? Is it by the motion of the blood, or by the bruit musculaire, or by any other concurrent alteration of the arrangement of the heart or of its contents? Let us first look at the opinions that have been advanced with respect to the production of the first sound. We must again quote freely from our author.

"The following causes (he says) have been severally assigned as physically capable of generating the first sounds during the systole of the ventricles. 1. The collision of the particles of fluid in the ventricles (Dr. Hope, Dr. Spittal.) 2. The rush of blood into the great arteries (Mr. Carlile.) 3. The closing of the mitral and tricuspid valves (M. Rouanet, Mr. Bryan.) 4. The muscular contraction itself.

1. The first of these explanations is ingeniously proposed by Dr. Hope, but he advances no facts in direct proof of the hypothesis. In a number of experiments which I have made on the generation of sound, I have found liquids, of all bodies, the most difficult to excite to sonorous vibration; and although they readily transmit vibrations already produced in solids, it requires a combination of circumstances to make them originate sound. This is consistent with the explanation which I have given of the production of sound (p. 5 *et seq.*), for impulses which throw solids into sonorous vibrations, are expended in liquids in causing a displacement of their particles. On making an experiment with a gum-elastic bottle, by filling it with water, and then forcibly compressing it under water by the end of the stethoscope (avoiding the use of the hand, for that produces its own muscular sound), I have failed in producing any sound at all approaching to that of the heart's contraction. The blood yields readily to the contracting ventricle, and there being no obstacle to the escape of blood from it, further than the weight of the arterial column, which the normal action of the heart can quietly and steadily overcome, it passes into the arteries without vibration. But if there be an obstacle to the current of the blood from the ventricle, whether that obstacle be a narrowing or a projection in the orifice, the current will act on it just as the bow does on the string of a violin; a sound will be excited, and thus are produced valvular murmurs. Again, if instead of the orifices being narrowed, the heart contracts with unnatural briskness, expelling its contents with convulsive energy, the natural outlets then become relatively narrow, and are thrown into vibrations; this is the *rationale* of the bellows murmur which accompanies the jerking pulse of pericarditis and the irritation of inanition. But the difference of these sounds, and of the circumstances that excite them, from those of the normal action of the heart, makes me hesitate to refer the latter to the same principle; and the fact that the morbid are often superadded to the natural sounds, also inclines me to think that they have a distinct cause.

2. The second explanation of the first sound, the rush of blood into the larger arteries, is perhaps less liable to the acoustic objection before urged, than the preceding opinion, for the blood has acquired an impulse when it enters the arteries, and if its course there is not free, it might readily produce a sound. But in their natural state, the arteries give passage to the blood as smoothly as the heart parts with it, and it would prove an imperfection in nature were it otherwise. Moreover, if this explanation were true, the larger arteries rather than the heart would be the principal seat of the sound; and the sound should be increased by an hypertrophied heart, with a strong pulse, and diminished by a dilated heart and a weak pulse; yet the reverse of these is presented in nature.

3. The closing of the auricular valves. The principal objection to this as the only cause of the first sound, is, that it must be instantaneous, and confined to the first part of the ventricular systole, whereas we know that the first sound is prolonged during the whole period of this action.

4. Although Laennec referred the first sound to the systole of the ventricles, he did not attempt to define the physical cause of its production. In the former edition of this work, I ventured to class it among the muscular sounds which Dr. Wollaston* first noticed to occur in all cases of rapid muscular contraction. This sound may be exemplified by applying the fleshy part of the thumb to the stethoscope or naked ear, and bending and straightening the thumb. It is louder in muscles that are thin, and in a state of considerable tension; and it is remarkable that it does not cease with the apparent movement, but continues as long as the muscle remains contracted and tense; it then takes on an intermitting character, like the noise of the rolling of a carriage over rough pavement, whence Dr. Wollaston was led to infer that muscular action is not perfectly continued, but consists of a series of minute contractions and relaxations. A good example of it may be obtained on applying the stethoscope to the neck of a person who holds his head back towards the opposite side, and then throws the platysma myoides into contraction. It still appears to me, that the most simple and satisfactory way of accounting for the first or systolic sound of the heart, is to refer it to this class of sounds. Their physical production seems to depend on the sudden tightness or tension into which the fibres of muscles are

* Croonian Lecture, Phil. Trans. 1810.

thrown when they contract; and the self-acting power of these fibres constitutes them the motors as well as the subjects of sonorous vibrations. Here we have to remark the extreme facility with which the motions of solids produce sounds, compared with those of fluids; for it is almost impossible to touch, stretch, bend, or compress solids, without throwing them into sonorous vibrations. In this case, we have a series of cords or fibres brought by a zig-zag tension, into a state of rigidity capable of sonorous vibrations, and the impulses developing these vibrations are communicated by the oscillations of those contracting fibres themselves; these oscillations in a greatly magnified degree, are apparent in the quivering seen and felt in the muscles of a horse drawing with unusual effort. The varieties observed in the contractions of the heart seem to me to be perfectly explicable on this principle. The sound begins the moment the fibres arrive at a state of tightening or tension; it continues until the contraction is completed and the blood expelled from the ventricle, and ceases the instant of the diastole. To perceive more readily the effect of hypertrophy, and of dilatation, let us attend to the sounds produced by the tension of linen or canvass (for muscle is, mechanically speaking, equally a web of fibres), and we shall find that in proportion as we thicken the substance, we obscure the sound which is produced on briskly stretching it; but when we use thin and simple webs, the sound becomes proportionally loud and clear. I shall not pursue the illustration of this explanation further, for I introduce it here only interrogatively, as deserving a place among other views, on the claims of which, future observation and experiment must decide. I must only remark, that M. Pigeaux is in error when he maintains that muscular sounds cannot be produced under water: I find them more distinct and free from adventitious sounds of the surface, and I have been able to imitate the sounds of the heart very exactly by muscular movements of the hand under water. I will conclude with the question, if the first sound of the heart is produced by another cause, what becomes of the muscular sound in this case of rapid muscular contraction?" 168.

So much for the theories of the immediate cause of the first sound. Prior to adverting to the experiments we shall speedily have to consider, we must state the two principal opinions which are entertained, in reference to the efficient cause of the second sound. They are, 1, The re-action of the arterial columns of blood against the semilunar valves. 2, The impulse of the blood from the auricles re-filling the ventricle at its diastole.

Such was the general state of the question, when the series of experiments we are now to notice were undertaken by Drs. Hope and Williams. When we commenced this article, we were not in possession of an Appendix just published by the former gentleman, in a second edition of his work on Diseases of the Heart.* That Appendix contains, as does Dr. Williams' book, the series of experiments in question. The account is substantially the same in the publications of both gentlemen. Having hitherto made use of that of Dr. Williams, we shall take from it also the particulars of the experiments, and add the conclusions drawn by each gentleman.

" *Experiment* 1.

About twenty grains of Woorara, moistened with water, having been inserted

* A Treatise on Diseases of the Heart, &c. &c. By J. Hope, M.D. F.R.S. &c. Second Edition, with an Appendix of Experiments, corroborating the previous series, and elucidating the causes of the sounds. 1835.

in an incision in the haunch of a donkey two months old, the animal died in fifteen minutes. Artificial respiration was immediately established, and the chest being opened, the pericardium was slit down and the heart exposed. Its pulsations were regular and vigorous, the auricles contracting immediately before the ventricles. The double sound was distinctly isochronous with the systole and diastole of the ventricles. The following points were then observed and noted, after repeated examinations by several present.*

Observation 1. The 1st sound was equally audible on all parts of the ventricles.

Obs. 2. The 2nd sound was most distinct near the roots of the great arteries, being audible there in the weaker pulsations, when it could not be heard by the stethoscope applied to other parts of the ventricles.

Obs. 3. Pressure on the arterial roots by the fingers, or by the stethoscope, invariably stopped the 2nd sound. Slight pressure caused a whizzing, or bellows murmur, with the 1st sound.

Obs. 4. On pushing the auricles, by the end of a finger, into each auriculo-ventricular opening, the ventricular contractions became weak and irregular; but the 1st sound although weak, was still heard alone.

Obs. 5. At each systole, the sudden tension or tightening of the ventricles was felt, by the finger applied to their body, as an abrupt shock, with which the 1st sound exactly coincided.†

Obs. 6. The left auricle was cut open, and the mitral valve partially destroyed; the blood issued in jets at each ventricular systole; yet the 1st sound still accompanied the systole. The 2nd sound was not heard after this incision.‡

Obs. 7. The right auricle was also freely laid open; still the 1st sound continued.

Obs. 8. I pushed my finger through the mitral orifice into the left ventricle, and pressed on the right, so as to prevent the influx of blood into either ventricle; the ventricles continued to contract strongly (especially when irritated by the nail of the finger in the left), and the 1st sound was still distinct, but not so clear as when the ventricles contracted on their blood.

Obs. 9. The same phenomena were observed when both the arteries were severed from the heart.

Until the auricles were cut open (as mentioned in 6 and 7), the 2nd sound was audible in all the strong pulses of the heart, but it was not heard after, although upwards of 30 pulses, most of them vigorous, took place. Ten or twelve

---

* "The following gentlemen were present; and I here beg to offer to them, and to those who assisted at the other experiments, my thanks for their kind co-operation and testimony.—Dr. Arnott, Author of the Elements of Physics, &c.; Mr. Babington, Surgeon to St. George's Hospital, &c.; Mr. Good, Surgeon to St. George's and St. James's Dispensary; Dr. Hope, Assistant Physician to St. George's Hospital, &c.; Mr. Henry Johnson, Demonstrator of Anatomy, &c.; Dr. J. Peregrine, House Surgeon at St. George's Hospital; Mr. G. Smith, Lecturer on Anatomy, &c.; Mr. Tatum, Lecturer on Anatomy, &c.

To Mr. Tatum, Mr. Henry Johnson, and Mr. H. James Johnson, I am especially obliged, not only for their able assistance, but also for the use of their splendid new dissecting-room, and its commodious appurtenances, in Kinnerton Street, where these experiments were performed."

† "This observation was, I think, suggested by Dr. Hope."

‡ "The results after 6 were witnessed by Dr. Hope, Mr. H. Johnson, and myself, the other gentlemen having left. The observations were noted during or immediately after the experiments; generally by Dr. Hope or myself."

strong contractions occurred after the introduction of the finger, as mentioned in Obs. 8.

This experiment lasted an hour and twenty minutes from the commencement of artificial respiration.

*Experiment* II.

About fifteen grains of Woorara (powdered, and made into a paste with water) were introduced into a wound under the haunch of a young ass, about six weeks old. The animal died in about thirty-five minutes. Artificial respiration was then immediately established, and the chest opened by cutting through the cartilages of the ribs, to the left of the sternum; and along the upper margin of one of the ribs near the shoulder, and then breaking back three or four ribs, so as to expose the contents of the left side of the chest. The following points were noted, as observed by several present.*

Obs. 1. Before the pericardium was opened, the 1st and 2nd sounds were very distinctly heard, although the heart touched no part of the parietes of the chest.

Obs. 2. Both sounds were distinctly heard through a lobe of the lung, interposed between the heart and the stethoscope.

Obs. 3. The pericardium being completely slit open, the 2nd sound was observed to be, decidedly, most distinct at the origin of the pulmonary artery and aorta, where it was louder than the 1st sound, and had perfectly its natural short, clear, flapping character. With the stethoscope applied on the body of the ventricles, the 2nd sound was heard less distinctly, and seemed more obtuse and distant.

Obs. 4. When the stethoscope was applied to the aorta about three inches from its origin, the 2nd sound (without the 1st) was heard following the systole of the ventricles as felt by the observer's finger.†

Obs. 5. The aorta and pulmonary artery being for a few seconds compressed between the finger and thumb, the 1st was accompanied with a bellows murmur, and the second sound ceased, during the continuance of the compression. This experiment was repeated several times by Dr. Hope and myself.

Obs. 6. A common dissecting-hook was passed into the pulmonary artery, and was made to draw back, and thus prevent the closure of the semilunar valves: the 2nd sound was evidently weakened, and a hissing murmur accompanied it. A shoemaker's curved awl was then passed into the aorta, so as to act in the same way on the aortic valves: *the 2nd sound now entirely ceased, and was replaced by a hissing.*

Obs. 7. The hook and the awl were withdrawn; *the 2nd sound returned, and the hissing ceased.* This and the preceding experiment were repeated, and observed by Dr. Hope, Mr. H. Johnson, Mr. Malton, and myself.

Obs. 8. Experiment 6 was repeated, with the same result, and whilst Dr. Hope listened, I withdrew the awl from the aorta. He immediately said, 'Now I hear the second sound.' I then removed the hook from the pulmonary artery; Dr. H. said, 'Now the second sound is stronger, and the murmur has ceased.'

Obs. 9. The pulmonary artery was cut open, and the finger introduced into the right ventricle;—the heart continued to contract irregularly, and the 1st sound alone was obscurely audible.

---

* Mr. Bushel, Lecturer on Anatomy, &c.; Mr. Good, Surgeon to St. George's Dispensary, &c.; Dr. Hope; Mr. H. Johnson, Surgeon, &c.; Mr. Keate, Surgeon to their Majesties, &c.; Dr. Macleod, Physician to St. George's Hospital, &c.; Dr. Page; Mr. Partridge, Junior Professor of Anatomy at King's College, &c.; Mr. Malton and Mr. Seagram, Pupils at St. George's Hospital; Mr. Willesford, Surgeon, &c."

† "This observation was suggested by Mr. Keate, and Obs. 2 by Dr. Hope.

Obs. 10. Slight contractions took place after the ventricles were laid open, and the columnæ carneæ were seen to contract simultaneously with the fibres of the ventricles.

These observations lasted during an hour and ten minutes after the commencement of artificial respiration; and until the opening of the artery in Obs. 9, the contractions of the heart were generally regular and vigorous." 174.

Such are the experiments, and we will now add severally the conclusions of Dr. Williams and of Dr. Hope. We shall then have put our readers in possession of the whole of the present question, its bearings, and its merits. And first then let us present the sentiments of Dr. Hope—then those of Dr. Williams.

"These experiments," says the former gentleman, "appear to warrant the following conclusions :—

A. The first sound is loudest on the body of the ventricles (1).

B. The second sound is loudest over the sigmoid valves, and thence for a few inches along the aorta (2, 12, 13).

C. Preventing the reaction of the blood on the sigmoid valves, annihilates the second sound (3, 14).

D. Creating regurgitation through the sigmoid valves occasions a murmur and extinguishes the second sound (15, 16, 17, 18).

From the two last propositions it results that the closure of the sigmoid valves is the cause of the second sound.

E. The jerk (impulse) felt on the ventricles is coincident with and occasioned by the closure of the auriculo-ventricular valves, by which a sudden resistance is offered to the ventricular contraction (5): under these circumstances the first sound is perfectly loud and distinct (1, 10).

F. When the resistance of the valves is removed, and the jerk thus prevented, the first sound is dull and obscure, (7, 8, 9, 19,) like the muscular sound which may be imitated with the hand.

The two last propositions seem to warrant the inference that the first sound is compound, viz. consisting, 1st, *possibly* of a degree of valvular sound, 2nd, of a loud, smart sound produced by the abstract act of sudden jerking extension of the muscular walls, in the same way that each sound is produced by similar extension of the leather of a pair of bellows—to avoid circumlocutions, I shall call this the *sound of extension*; 3rd, a prolongation, and possibly an augmentation, of this sound by the sonorous vibrations peculiar to muscular fibre (*bruit musculaire, muscular sound*).

These heads will severally require a few remarks. The *valvular sound* can only be spoken of as *possible*, because being synchronous with, and as it were incorporated in, the sound of extension, its existence is not demonstrable. The *possibility* may perhaps amount to a *probability*, on the principle that, if the sigmoid valves can produce sound, the same may, by analogy, be predicated of the auriculo-ventricular valves.

The existence of the *sound of extension* appears to me to rest on strong grounds. The phenomena in No. 5 made a forcible impression on all present; and it was remarked that the sense of touch conveyed an identical idea with the sense of hearing. The first sound of the heart during palpitation is, in some instances, of such extraordinary intensity, that it would do violence to all analogy to suppose it produced solely by *bruit musculaire;* and some of the strongest advocates for this *bruit* have thought it necessary, in such cases, to imagine a sound produced extrinsically, by the heart impinging against the thoracic walls. This corroborates the opinion which I formerly expressed, (page 47,) founded upon, and subsequently confirmed by, every variety of experiment on muscular sound which I could devise. Nor can it be supposed, again, that the auriculo-ventricular valves alone could produce so loud a first sound as is sometimes heard; not to say that this sound is of a different character, being much more blunt than the loudest valvular click, taking that of the sigmoids as a type.

The *bruit musculaire* appears to constitute the prolongation of the first sound, this prolongation being of the same dull, rumbling character, as ordinary muscular sound. Possibly it may add to the intensity of the first sound. Nor can it be affirmed that the motion of the blood does not also contribute to the sound, though there is no proof that it does. In the healthy human subject, when faint, the first sound is short and flapping, like the second. For this, *bruit musculaire* alone would not account, as languor would increase the characteristic dulness and obscurity of the *bruit musculaire.* The first sound during faintness, therefore, must proceed from muscular extension, or from the valves, or from both."—*Appendix,* vii.

Dr. Williams's conclusions follow.

"The deductions," says Dr. W., "from these results are simple and obvious, and they not only decide between the views before stated, but they appear to me to demonstrate most satisfactorily the true seats and causes of the sounds of the heart.

That the first sound is not caused by the rush of blood into the great arteries, (as supposed by Mr. Carlile) is proved by Obs. 4, 6, 7, 8 and 9, of Exper. I.; and Obs. 9 of Exper. II.; in which the first sound continued, although little, and in the latter cases no blood, could have been thrown into the arteries. A further proof against this view may be seen in Exper. II. Obs. 4, from which it appears that the first sound is much less audible in the large arteries than in the heart.

That the 1st sound is not dependent on the closing of the auriculo-ventricular valves, (as imagined by M. Rouanet and others) is evident from Exper. I. Obs. 4, 6, 7, 8, 9, in which the closure of these valves was partially or completely prevented, yet the 1st sound still continued.

That the first sound is not produced by the collision of the particles of fluid in the ventricles, (as formerly conceived by Dr. Hope,) appears from Obs. 4, 8, and 9, of Exper. I.; and Obs. 9 of Exper. II.; in which the sound was produced, although there was no blood in the ventricles.

*That the 1st sound is produced by the muscular contraction itself,* may be considered as proved by Obs. 8 and 9 of Exper. I.; in which every other possible source of sound was excluded, and the 1st sound still accompanied the systolic action of the ventricles.*

*That the 2nd sound is produced by the reaction of the arterial columns of blood tightening the semilunar valves at the ventricular diastole,* is clearly proved, not only by a situation of these valves being the special seat of the sound, (Exp. I. Obs. 2; and Exp. II, Obs. 3,4) but also by the numerous observations in which the cessation or reproduction of the sound was effected by the suspension or restoration of the action of these valves. (Exp. I. Obs. 3; Exp. II. Obs. 5, 6, 7, 8.)

It being thus proved that the 1st sound is essentially produced by the tight-

* "This view of the cause of the 1st sound, was first published by me in the first edition of this work, in 1828, and I am not aware that it has been entertained by any other writer. M. Majendie is in error when he ascribes a similar opinion to Laennec, who, on the contrary, only associated the abnormal murmurs with the muscular sound produced by a fancied spasm of the heart and arteries; but neither in his works, nor in his lectures, did he give any opinion as to the physical causes of the healthy sounds of the heart."

ening of the muscular parietes of the ventricles, and the 2nd sound by the subsequent sudden tension of the semilunar valves, it is easy to perceive how various circumstances may increase or diminish the sounds, as they augment or impair the degree or abruptness of this tightening or tension in these parts. Thus the mass of blood in the heart increases the clearness of the 1st sound, by affording an object, around which the fibres effectually tighten; whilst the auricular valves, by preventing the reflux of this blood, increase its resistance, and thus add to the tension necessary for its expulsion. Probably, in common pulsations, the ventricles do not attain the degree of tension which is sonorous until the closing of the auricular valves; this closure, as the commencement of the resistance, brings at once to its acmé the muscular tension, which continues until the contents of the ventricles are sufficiently expelled. This accounts for the sudden or flapping commencement often perceptible in the 1st sound, and it suggests how the due action of the auricular valves generally contributes to its clearness. The degree and abruptness of the systolic tension was well seen in Obs. 5, of Exp. I.; and the external feel and view of the systole gave us the impression of its being a motion in itself sufficient to produce sound.* The auricular valves, the cordæ tendineæ, the columnæ carneæ, and internal fibres of the ventricles, if they attain the same degree of tension as the exterior of the ventricles, may have an equal share in the production of the 1st sound; but I am disposed to think, that what we hear proceeds chiefly from the contracting tenseness of the external walls of the heart, both because they are nearer to the ear, and because in Exper. I. Obs. 8, the contraction of the left ventricle upon my finger within it, was by no means so abrupt or strong as that of the exterior as felt by the other hand, and still heard through the stethoscope.

The termination of the systole of the ventricles is abrupt, being immediately followed by the diastole: and it is obvious that the first effect of this must be, to occasion the sudden closing of the semilunar vales, pressed now only on their concave side by the force of the arterial column of blood. Hence the 2d sound immediately succeeds the 1st, or rather appears to terminate it, by its abrupt, clear *flap*, which in the healthy heart differs as much in character from the 1st sound, as the simple valves by which it is produced differ from the thicker muscular web of the ventricles, the tightening of which causes the 1st sound. The mobile state of these valves, the bulk of blood propelled by the ventricular systole into the arterial column, and the suddenness of the diastole by which this column is brought to press fully backwards on the valves, are the circumstances which give clearness and loudness to the 2nd sound; and it may be seen, in the foregoing experimental observations, how various causes interfering with these, impaired or suspended the 2nd sound. Exp. I. Obs. 3, 4, 6, &c. Exp. II. Obs. 5, 6, 7, 8, 9." 178.

Such are the respective deductions drawn from the same experiments by the gentlemen before us. It remains for our readers to determine the degree of value, that they can attach to conclusions based upon premises of this description. There cannot be one rational doubt that the first sound and the impulse are immediately dependent on the ventricular contraction, and that they become, pathologically speaking, its guage and measure. This is an important fact, enabling us, as it does, to determine by a plain and exact physical sign, the condition of the ventricle. And, as diseases of

* "The results of these experiments so completely confirm the views that I stated in the last edition of this work with respect to muscular action as a cause of sound, that I need here only refer to my former remarks as quoted in the note, p. 167."

the ventricles, especially the left, are perhaps the most frequent in the series of affections of the heart, we need scarcely point out the value of any symptom or sign which brings an addition of certainty to their diagnosis. Had auscultation done only this, it would still have conferred an essential benefit on medicine.

But what shall we say to the present investigations on the nature of the second sound? The experiments conducted by Dr. Hope and Dr. Williams, are certainly ingenious, and may seem satisfactory. They may seem satisfactory, but they are not demonstrative, and the amount of satisfaction must depend as much on the temper of mind with which they are regarded, as on their intrinsic exactness and freedom from liability to error. It may fairly be assumed in reference to experiments of this description, that the simpler and the less complicated they are made, the more likely they are to lead to determinate results. An acquaintance with the history of experimental physiology is not calculated to increase our stock of faith, nor to abate that scepticism which is Reason's shadow. Rational philosophical scepticism is friendly not inimical to truth; accelerates, and never can retard its progress. That scepticism in science we profess without shame, and we trust that it does not degenerate into prejudice.

These remarks may appear apologetic, and they are so—apologetic for the doubts we are about to hint with respect to the conclusiveness of the present investigations. Those doubts will be found to be essentially based on the principle to which we have already alluded—that the farther we depart from simplicity in experiments, the nearer we approach to fallacy in the results.

A glance at the pages over which we have travelled, with interest, if not with speed, will enable us to perceive that the following circumstances have led Dr. Hope and Dr. Williams to the conclusion that the second sound is occasioned by the closure of the sigmoid valves:—first, the second sound was more distinctly heard over the origins of the aorta and the pulmonary artery, than on the body of the ventricles; and, secondly, hooks passed into the pulmonary artery and the aorta, in such a way as to prevent the closure of the sigmoid valves, destroyed the second sound, and substituted for it a prolonged hissing, which hissing disappeared while the second sound returned, on removing the hook from the vessels in question. Such are the two facts which appear to establish the induction of our authors, that the second sound depends on the closure of the sigmoid valves. Let us see if any portion of fallacy can stick to them.

The anatomist is aware that the *origins* of the pulmonary artery and the aorta, and more particularly that of the latter, are concealed in the body of the ventricle. How nice then must that discrimination be, which, by the ear, amidst the bustle and confusion of an experiment, can determine with accuracy differences of sound dependent on differences of situation so minute. The aorta arises from the upper part of the left ventricle, the semilunar valves are situate precisely at its origin, a considerable portion of the substance, we may say, of the base of the ventricle intervenes between the cause of the sound (those valves) and the spot on which the stethoscope is placed, yet the latter distinguishes, or is considered to distinguish, the modifications of sound occasioned by *such* varieties of situation. We do not push this indirect objection farther than it ought fairly to be carried.

It does not overthrow the argument, but it certainly tends to render it unsatisfactory.

It may appear that the second is the stronger fact—that the total destruction of the sound, by prevention of the action of the semilunar valves, is conclusive with respect to the dependence of the one upon the other. Yet here again a rational scepticism finds room and necessity for doubt. In the first place, the experiments are not adequate in point of number to the proving of the induction, proving it, we mean, any thing approaching demonstration. In the second place, it may be questioned whether the semilunar valves were really prevented from acting by the introduction of a hook in the manner described by the experimenters. For, let any one endeavour to do this leisurely and with every facility that quiet and the absence of hurry can afford, on the heart of the dead animal; he will find that he cannot with certainty effect it. In the third place, there must necessarily be much violence inflicted, much disturbance produced, by attempts of this description—disturbance of a serious character when the object is to determine the causes of sound. If the action of the semilunar valves gives rise to a definite and audible sound, surely the passage of hooks into the great arteries, and rudely holding or tearing those valves, should occasion some acoustic phenomena, some physiological confusion.

Such are the doubts, the natural doubts, that a perusal of these papers creates in our minds. They are merely doubts, it is true, and possibly they may be shewn to be groundless and hypercritical. We shall feel great pleasure at finding that they are so; but we repeat that philosophical scepticism is absolutely indispensable if we wish to discover truth.

